# The Dietary Inflammatory Index Is Positively Associated with Colorectal Cancer Risk in a Chinese Case-Control Study

**DOI:** 10.3390/nu12010232

**Published:** 2020-01-16

**Authors:** Alinuer Abulimiti, Xin Zhang, Nitin Shivappa, James R. Hébert, Yu-Jing Fang, Chu-Yi Huang, Xiao-Li Feng, Yu-Ming Chen, Cai-Xia Zhang

**Affiliations:** 1 Department of Epidemiology, School of Public Health, Sun Yat-sen University, Guangzhou 510080, China; 2 Guangdong Provincial Key Laboratory of Food, Nutrition and Health, School of Public Health, Sun Yat-sen University, Guangzhou 510080, China; 3 Cancer Prevention and Control Program, University of South Carolina, Columbia, SC 29208, USA; 4 Department of Epidemiology and Biostatistics, University of South Carolina, Columbia, SC 29208, USA; 5 Connecting Health Innovations LLC, Columbia, SC 29201, USA; 6 Department of Colorectal Surgery, State Key Laboratory of Oncology in South China, Collaborative Innovation Center for Cancer Medicine, Sun Yat-sen University Cancer Center, 651 Dongfeng Road East, Guangzhou 510060, China; 7 Department of Experimental Research, State Key Laboratory of Oncology in South China, Collaborative Innovation Center for Cancer Medicine, Sun Yat-sen University Cancer Center, 651 Dongfeng Road East, Guangzhou 510060, China

**Keywords:** diet, inflammation, dietary inflammatory index, colorectal cancer risk, case-control study, China

## Abstract

Diet may modulate chronic inflammation. The aim of this study is to investigate whether the dietary inflammatory index (DII^®^) was associated with the risk of colorectal cancer in a Chinese population. A case-control study was conducted from July 2010 to April 2019, in Guangzhou, China. A total of 2502 eligible cases were recruited along with 2538 age- (5-year interval) and sex-matched controls. Dietary data derived from a validated food frequency questionnaire were used to calculate the energy-adjusted DII (E-DII) scores. Odds ratios (ORs) and 95% confidence intervals (CIs) for colorectal cancer risk were estimated using unconditional logistic regression models. In this study, E-DII scores ranged from −5.96 (the most anti-inflammatory score) to +6.01 (the most pro-inflammatory score). A positive association was found between the E-DII and colorectal cancer risk, with the OR = 1.40 (95% CI 1.16, 1.68; *P*_trend_ < 0.01) for the highest E-DII quartile compared with the lowest quartile after adjusting for potential confounders. When stratified based on cancer subsite, sex, body mass index, and smoking status, significant associations were not observed in women or underweight individuals. Results from this study confirmed that a higher E-DII score was associated with an increased risk of colorectal cancer in a Chinese population.

## 1. Introduction

Colorectal cancer is a common cancer around the world, with 1.8 million new cases estimated to have occurred worldwide in 2018 [1]. Chronic inflammation plays a critical role in colorectal carcinogenesis [2]. Evidence suggested that inflammation promotes cancer cell growth, inhibits differentiation, and reduces survival through a complicated set of molecular pathways, including the release of a large number of pro-inflammatory cytokines such as interleukin (IL)-1, IL-6, IL-8, and tumor necrosis factor-α (TNF-α) [2,3]. It is also known that patients with inflammatory bowel diseases are at increased risk of developing colorectal cancer [4,5], and anti-inflammatory treatment, especially prolonged use of nonsteroidal anti-inflammatory drugs (NSAIDs) could reduce the risk of colorectal cancer by as much as 30 to 40% [6].

Dietary factors may modulate chronic inflammation [7,8]. For example, the western-type diet, which is high in red meat, fat, and refined grains is pro-inflammatory [9]. By contrast, the Mediterranean diet, which is characterized by a high consumption of vegetables, fruits, and whole grains, appears to mitigate the degree of inflammation [10] and is associated with decreased risk of colorectal cancer [11]. Therefore, assessing the potential effect of diet on inflammation may help to inform dietary strategies to decrease the levels of inflammation and risk of colorectal cancer. 

The dietary inflammatory index (DII^®^) is a literature-derived, population-based scoring system that was developed to quantify the inflammatory potential of overall diet in diverse populations [12]. A higher DII score represents a more pro-inflammatory diet, while a lower DII score indicates a more anti-inflammatory diet. The DII has been validated with inflammatory markers, such as C-creative protein (CRP), IL-1, IL-2, and TNF-α [13,14,15,16,17,18,19]. 

To date, five prospective cohort studies [20,21,22,23,24] and nine case-control studies [25,26,27,28,29,30,31,32,33] have investigated the association between the DII and colorectal cancer risk, and all of them have found that a higher DII score is associated with an increased risk of colorectal cancer. Four [30,31,32,33] of nine case-control studies were conducted in Asian countries, including South Korea, Jordan, and Iran. However, to the best of our knowledge, no study has been conducted in Chinese population, whose socio-demographic characteristics and dietary patterns that are characterized mainly by higher consumption of vegetables, rice and fish/sea food vary from those of other populations [34]. Therefore, this study aimed to examine whether diet-related inflammation, as measured by the DII score, was linked to the risk of colorectal cancer in a Chinese population. Our hypothesis was that higher, pro-inflammatory, DII scores would be positively associated with increased colorectal cancer risk.

## 2. Materials and Methods

### 2.1. Study Subjects

This is an ongoing case-control study, which began in July 2010. The study protocol has been reported in detail elsewhere [35]. Briefly, potential cases were consecutively recruited from the surgical units of the Sun Yat-sen University Cancer Center, Guangzhou, China, from July 2010 to April 2019. To be eligible for this study, patients were required to have incident, primary, histologically confirmed colorectal cancer diagnosed no more than 3 months prior to the interview. Participants needed to be between 30 and 75 years of age and either be natives of Guangdong province or have lived in Guangdong province for more than 5 years. Patients were excluded if they had been diagnosed with any cancer other than colorectal cancer or could not understand or speak Mandarin/Cantonese. Out of 2817 eligible patients, 2528 agreed, resulting in a participation rate of 89.74%. In addition, 26 subjects were removed from the analysis because of implausible energy intakes (<600 or >3500 kcal/d for women, and <800 or >4200 kcal/d for men) [36]. Data from a total of 2502 patients were included in these analyses.

Controls, also 30 to 75 years of age but with no history of cancer, were frequency matched to cases on age (5-year interval) and sex. There are two control groups in this case-control study. The first control group was selected from patients who visited the first affiliated hospital of Sun Yat-sen University during the same period, with ear-nose-throat diseases, eye disorders, facial paralysis, orthopedic conditions, degenerate joint diseases, and varicose veins. A total of 1533 hospital-derived controls were invited in this study and 1357 were successfully interviewed, yielding a response rate of 88.51%. The second group of 1181 community-derived controls was recruited in Guangzhou and Shenzhen via referrals, invitation, and advertisements.

The study was conducted in accordance with the ethical standards laid down in the 1964 Declaration of Helsinki and its later amendments. The protocols and procedures of this study were approved by the Ethical Committee of School of Public Health, Sun Yat-sen University. All participants in this study signed the informed consent form before the interview.

### 2.2. Data Collection

A face-to-face interview was conducted by trained researchers using standardized questionnaires to collect data on socio-demographic characteristics, lifestyle factors (active and passive smoking status, alcohol drinking, physical activity), first-degree relatives with cancer, history of diabetes mellitus and inflammatory diseases (e.g., gastritis, hepatitis, arthritis), body height and weight. For women, information on menopausal status also was obtained. In this study, subjects were classified as nonsmokers and ever smokers (including regular smokers and former smokers). A regular smoker is defined as someone smoking at least 1 cigarette per day for more than 6 consecutive months, while former smokers are defined as those who reported being regular smokers in the past, but not having smoked in the past six months. Passive smoking is defined as being exposed to the tobacco smoke of others for at least 5 min per day during the previous 5 years. Regular drinkers are defined as subjects who drank alcohol at least once per week in the past year. Physical activity consisted of two parts: occupational activity and household and leisure-time activity. Occupational activity was grouped into nonworking, sedentary, light occupation, moderate occupation and heavy activity occupation according to labor intensity. Household and leisure-time activity is classified as follows: (a) light physical activity; (b) moderate physical activity; (c) vigorous physical activity [37]. Information on frequency (day/week) and duration (hour/day) of household and leisure-time activity was collected. The mean metabolic equivalent task-hours (MET) score of each activity was obtained by estimating the average of all comparable activities in the Compendium of Physical Activities [38,39]. MET-hours/week over the past year was calculated as follows: number of days per week × number of hours per day × MET for a specific type of activity = MET-hours/week. History of diabetes mellitus and inflammatory diseases were defined by self-report of physician diagnosis or from medical records. Body weight was measured and height was self-reported. Body mass index (BMI) was calculated by dividing weight (kg) by height squared (m^2^). All subjects were categorized into three groups based on BMI range of Chinese population: underweight (BMI < 18.5 kg/m^2^), normal weight (18.5 kg/m^2^ ≤ BMI < 24 kg/m^2^), overweight/obese (BMI ≥ 24 kg/m^2^) [40].

A reproducible and validated food frequency questionnaire (FFQ) was used to assess the participants’ daily consumption of various foods [41]. Detailed information was collected on frequency of consumption and portion size during 12 months prior to colorectal cancer diagnosis for cases and interview for controls. The 81-item FFQ included 12 cereal items, 7 legume items, 18 items of vegetables, 11 fruit items, 18 meat (read meat and white meat) items, 2 egg items, 8 dairy product items, 3 questions on beverages and soups, and 2 questions on mushrooms and nuts. Intakes of total energy and nutrient were determined using the Chinese Food Composition Table [42].

### 2.3. Dietary Inflammatory Index (DII^®^)

Dietary data derived from the FFQ were used to calculate the energy-adjusted dietary inflammatory index (E-DII) scores. The development of the DII has been described in detail elsewhere [12]. In short, 1943 articles published through 2010 on the relationship between 45 food parameters and six inflammatory biomarkers (IL-1β, IL-4, IL-6, IL-10, CRP, and TNF-α) were reviewed and scored. A world database from 11 countries was used to standardize DII/ E-DII scores to its current range and to provide reference means and standard deviations of intake for food parameters. Then a Z-score was calculated for each participant by subtracting the world standard mean from the actual intake value and dividing by its world standard deviation. In order to minimize the effect of right skewing, this Z-score was converted to a proportion. The proportion was then centered on one with a range −1 to +1, by doubling and subtracting 1. Then this value was multiplied by the inflammatory effect score for each of up to 45 food parameters and summed across all food parameters to create the overall dietary inflammatory score. The score ranged from 7.98 (the most pro-inflammatory score) to −8.87 (the most anti-inflammatory score). In a manner analogous to that used for the DII, energy-adjusted DII (E-DII) scores were calculated per 1000 kcal consumed to control for the effect of energy intake. This required using the same procedure for the DII, except the reference database that also was adjusted per 1000 kcal.

For this study, the following 34 food items were used for calculating E-DII scores: carbohydrate, protein, total fat, fiber, saturated fat, monounsaturated fatty acid (MUFA), polyunsaturated fatty acid (PUFA), n-3 PUFA, n-6 PUFA, cholesterol, thiamin, riboflavin, niacin, folic acid, vitamin B6, vitamin B12, vitamin A, vitamin C, vitamin D, vitamin E, β-carotene, iron, magnesium, selenium, zinc, flavan-3-ol, flavone, flavonols, flavonones, anthocyanidins, isoflavones, garlic, onion, and pepper.

### 2.4. Statistical Analysis

All analyses were performed using SPSS^®^ 22.0. Differences between cases and controls were assessed using the Student’s t test or the Wilcoxon rank-sum test for continuous variables, and the χ^2-^test for categorical variables. The Kruskal–Wallis test for continuous variables was used to examine intakes of 34 food parameters according to quartile of the E-DII score. The E-DII was analyzed both as a continuous variable and a categorical variable; i.e., as quartiles based on the distribution in controls [43]. The lowest quartile of the E-DII score was treated as the reference. Odds ratios (ORs) and 95% confidence intervals (CIs) were estimated using unconditional logistic regression models to explore the relationship between the E-DII and colorectal cancer risk. The following potential confounding factors were included in the model based on comparison of characteristics between cases and controls, or previous report: age, sex, marital status, residence (city/rural), educational level, occupation, income, BMI, smoking status, alcohol drinking, first degree relative with cancer, occupational activity, household, and leisure-time activities. Tests for linear trend were carried out by entering the categorical variable as continuous variable in the regression model. Stratified analyses were conducted for cancer site (colon cancer and rectal cancer), sex, BMI (underweight, normal weight and overweight/obese), and smoking status (ever smokers and nonsmokers). All tests were two-sided and the significance level (α) was set at 0.05.

## 3. Results

Of the 2502 cases, 1425 were men while 1077 were women; 1565 were diagnosed with colon cancer and 937 were diagnosed with rectal cancer. The socio-demographic characteristics of the subjects are shown in Table 1. Compared with controls, cases were more likely to be married, to live in the rural area, to have a lower BMI, be less educated, and have lower incomes. Cases also were more likely to have a first-degree relative with cancer, a history of diabetes mellitus, have heavier occupational activity and lower household and leisure-time activities, and to be ever smokers and regular drinkers. The difference between cases and controls was not significant for other variables, such as age, age at menarche, sex, menopausal status, passive smoking status, and history of inflammatory diseases. 

In this study, E-DII scores ranged from −5.96 (the most anti-inflammatory score) to +6.01 (the most pro-inflammatory score), and cases had a higher mean E-DII score (−0.50) compared with controls (−0.78). 

The distribution of 34 food parameters across quartiles of the E-DII among control subjects is presented in Table 2. Higher E-DII scores, indicating a more pro-inflammatory diet, were significantly related to lower intakes of fiber, cholesterol, folic acid, vitamin A, vitamin C, vitamin D, vitamin E, β-carotene, magnesium, selenium, flavan-3-ol, flavones, flavonols, flavonones, anthocyanidins, and isoflavones. Subjects in the fourth quartile of the E-DII had the highest consumption of carbohydrate, total fat, saturated fat, MUFA as well as PUFA. Median intakes of thiamin were equal in each quartile. Intakes of protein, n-3 fatty acids, riboflavin, vitamin B6, vitamin B12, iron, garlic, onion, and pepper in the first quartile were higher than those in other quartiles.

As shown in Table 3, a positive association was found between E-DII score and colorectal cancer risk, with an adjusted OR of 1.07 (95% CI 1.03, 1.11) when the E-DII score was fit as a continuous variable. A similar result was observed when modeling the E-DII score as quartiles. After adjusting for potential confounders, the OR for the highest quartile compared with the lowest quartile was 1.40 (95% CI 1.16, 1.68; *P*_trend_ < 0.01). Subgroup analysis by cancer site showed that diet-related inflammation is associated with increased colorectal cancer risk in both colon and rectal cancer, with adjusted ORs for the highest quartile compared with the lowest were 1.32 (95% CI 1.07, 1.63; *P*_trend_ = 0.01) for colon cancer, 1.53 (95% CI 1.19, 1.98; *P*_trend_ < 0.01) for rectal cancer, respectively.

Stratified analysis by sex indicated that the E-DII was significantly related to colorectal cancer risk only in men (OR_Q4 vs. Q1_ 1.74; 95% CI 1.35, 2.24; *P*_trend_ < 0.01). In women, a marginally significant positive association was found (OR_Q4 vs. Q1_ 1.28; 95% CI 0.98, 1.71; *P*_trend_ = 0.04). However, the interaction between the E-DII and sex in the risk of colorectal cancer was non-significant (*P*_interaction_ = 0.52). When stratified based on BMI, higher E-DII scores were associated with increased colorectal cancer risk among normal weight and overweight and obese participants. Adjusted ORs for the highest quartile of the E-DII score relative to the lowest were 1.32 (95% CI 1.02, 1.71, *P*_trend_ = 0.13) for persons with normal weight and 1.49 (95% CI 1.12, 1.97; *P*_trend_ < 0.01) for persons with overweight or obesity; no statistically significant result was found in underweight people (OR_Q4 vs. Q1_ 1.56; 95% CI 0.63, 3.87; *P*_trend_ = 0.28) (*P*_interaction_ = 0.03). The results of stratified analysis by smoking status were similar to the main analysis in all study subjects. A stronger positive association was found between the E-DII score and colorectal cancer risk among ever smokers and non-smokers, with adjusted OR of 1.74 (95% CI 1.26, 2.39; *P*_trend_ < 0.01) and 1.34 (95% CI 1.07, 1.68; *P*_trend_ = 0.01), respectively, comparing the highest with the lowest quartile. There was no statistically significant interaction between the E-DII and smoking status (*P*_interaction_ = 0.25) (Table 4).

## 4. Discussion

This large case-control study focused on the relationship between E-DII score and colorectal cancer risk. The results showed that a more pro-inflammatory diet, as reflected by a higher E-DII score, was associated with an increased risk of colorectal cancer. These findings persisted after adjusting for various confounding factors. 

Chronic inflammation might be causally linked to the development of carcinogenesis through complicated molecular pathways [2,3]. Previous studies have shown the modulating effects of dietary components on inflammation [7,8]. For example, red meat and fat play a pro-inflammatory role while fruit and vegetable have anti-inflammatory effects [8]. However, little is known about the inflammatory potential of whole diet, as most of studies focused on a single food or nutrient. Foods and nutrients are always consumed with other food groups and nutrients, thus dietary interactions may influence the real associations for diet with inflammation. The DII and E-DII were developed to assess the pro-inflammatory and anti-inflammatory effects of overall diet [12]. Previous studies have validated the close relationship between the DII/ E-DII and inflammatory markers [13,14,15,16,17,18,19]. 

The present study provides evidence regarding the association between a higher E-DII score and an increased risk of colorectal cancer in a Chinese population. In concordance with this result, previous epidemiologic studies observed significant positive association between the DII and colorectal cancer risk [20,21,22,23,24,25,26,27,28,29,30,31,32,33]. A 2017 meta-analysis of four prospective cohort studies and five case-control studies also reported an overall 40% increased risk of colorectal cancer in the highest quartile of the DII score [44], a result consistent with what we found. Additionally, one prospective study from the Iowa Women’s Health Study considered food parameters from both FFQ and supplements to create DII scores and showed that a higher DII score was linked to an increased risk of colorectal cancer when foods and supplements were both considered [20]. However, when analyses were performed with the DII from food groups only, the association became weaker and did not reach statistical significance in the highest quintile versus the lowest DII category (HR_Q5 vs. Q1_ 1.16; 95% CI 0.97, 1.69). 

There are several mechanistic explanations for the role of diet-related inflammation in colorectal carcinogenesis. First, the pro-inflammatory potential of diet may contribute to insulin resistance by increasing chronic inflammation [45], which can elevate levels of insulin, glucose, and triglycerides in vascular tissue to exert effects on cell cycle control, survival and cell mutation, thereby increasing colorectal cancer risk [46]. Another possible mechanism is through the activation of the cyclooxygenase-2 (COX-2) pathway to promote focal proliferation and mutagenesis in colonic epithelial cells [47].

There are differences between colon and rectal cancer in etiology and pathologic characteristics, as well as genetic abnormalities [48]. In the present study, analysis of cancer site-specific data produced similar results as main analysis in all colorectal cancer cases. In agreement with our results, a prospective study conducted in the United States reported that a higher DII score was linked to increased risk of both colon cancer and rectal cancer [23]. However, results from the Women’s Health Initiative indicated a significant positive association for the DII with respect to the risk of colon cancer, but not rectal cancer [21]. Further investigations are needed to clarify associations between diet-associated inflammation and colon and rectal cancer.

In the present study, the positive association between the E-DII and colorectal cancer risk was restricted to men. In women, we observed a similar tendency toward increased risk with higher E-DII scores, but results did not achieve statistical significance; perhaps due to reduced power because of smaller sample size. The National Institutes of Health-American Association of Retired Persons Diet and Health Study also found a statistically increased risk of colorectal cancer with a higher DII score in men, but not in women (OR_Q4 vs. Q1_ 1.44; 95% CI 1.29, 1.61 for men; and OR_Q4 vs. Q1_ 1.12; 95% CI 0.95, 1.31 for women) [22]. The difference that we observed by sex was not as dramatic and, just barely missed being statistically significant in women. Slight differences in the magnitude of the effect according to sex may be related to the mediating effect of estrogen on colorectal cancer and inflammation. A nested case-control study indicated that endogenous estrogen levels were inversely associated with colorectal cancer risk and suggested that endogenous estrogens may inhibit colorectal carcinogenesis through molecular pathways [49]. Based on in vitro studies, β estrogen receptor could down-regulate pro-inflammatory cytokines, such as IL-6, to play a colon cancer-prevention effect [50,51].

Stratified analysis based on BMI status showed that the positive association of the E-DII score with colorectal cancer risk was stronger in overweight and obese individuals as compared to normal-weight individuals, suggesting that the association may be reinforcing obesity-related inflammation. One of the possible mechanisms for the relationship between obesity, inflammation, and colorectal cancer risk can be explained by the release of inflammatory factors by adipose tissue-derived cell types, including macrophages and adipocytes [52]. Obesity may cause infiltration of adipose tissue by a large number of M1-polarized macrophages (a pro-inflammatory phenotype) that gradually replace M2-polarized cells (an anti-inflammatory phenotype) [53,54]. M1 macrophages lead to adipose secretion of several pro-inflammatory cytokines, such as TNF-α, IL-1, and IL-6, contributing to affect the microenvironment of tumor growth [55]. Furthermore, adipocytes also can produce polypeptide hormones (e.g., adiponectin and leptin), TNF-α, IL-6 and free fatty acid to mediate inflammatory response in colorectal cancer [56].

Smoking is associated with a wide range of alterations in inflammatory marker levels and smoking-induced inflammation may be a critical mechanism in cancer development [57]. In the present study, we observed a stronger positive association between the E-DII and colorectal cancer risk among ever smokers compared with nonsmokers. Few studies have explored the association based on smoking status. A study conducted in Germany found that diet-related inflammation had no effect on all-cause mortality in colorectal cancer survivors who smoked or did not smoke [58]. But those results should not be over-interpreted as there were only 126 current smokers among the 1404 colorectal cancer long-term survivors. 

The present study has several strengths. This is the first epidemiologic study to investigate the association between the inflammatory potential of diet and colorectal cancer risk in China, which has different dietary patterns and high colorectal cancer incidence. The sample size was relatively large relative to other case-control studies. Generally, study participants did not know the inflammatory potential of different foods, and the hypothesis of our research also was not revealed to all study subjects, thereby minimizing information bias. Moreover, several potential confounders, including socio-demographic characteristics and lifestyle factors, were adjusted when assessing the association between the E-DII and colorectal cancer risk.

This study also has some limitations. First, selection bias is potential limitation in case-control studies. All cases were recruited from Sun Yat-sen University Cancer Center, the biggest cancer center in Southern China. However, the clinical characteristics of colorectal cancer patients in this center were similar to those of patients from other large hospitals in Guangdong province [59] or in mainland China [60]. In addition, the high participant rate (89.74 and 88.51% for cases and hospital-derived controls, respectively) also served to reduce selection bias. Second, the possibility of recall bias should be considered. To minimize the bias, we interviewed newly diagnosed colorectal cancer patients and the average time interval between their diagnosed and the interview was 9.8 days. Photographs of foods with usual portion size also were provided for subjects to collect dietary information accurately. Third, 11 of the 45 food parameters were not available for calculating the E-DII score, including components such as turmeric, saffron, eugenol, rosemary, and thyme/oregano. It should be noted, however, that these components are infrequently consumed in Chinese adults; so, non-availability of these components may not have had a major impact. Also, a previous study showed that the statistical association was stable when only 28 food parameters out of 45 were used for the DII calculation [23]. Finally, our results were not adjusted for regular use of anti-inflammatory drugs, such as NSAIDs or Aspirin, which may mask the real relationship between the dietary inflammatory potential and colorectal cancer risk. However, we interviewed the cases as soon as the diagnosis and treatments were made, and most of these cases had not used NSAIDs or Aspirin before the interview. Therefore, the results of our study may not be greatly affected by regular use of some anti-inflammatory drugs. 

In conclusion, a higher E-DII score was associated with an increased risk of colorectal cancer among Chinese population, suggesting a potential role of diet-related inflammation in colorectal carcinogenesis. Results from this important study in a unique Chinese population add to the impressive list of others that previously examined the role of diet-related inflammation in colorectal carcinogenesis and are now part of the established literature on the subject.

## Figures and Tables

**Table 1:** Characteristics and selected risk factors of colorectal cancer cases and controls in a Chinese case-control study, 2010–2019 ^a^.

	Cases (*n* = 2502)		Controls (*n* = 2538)		*p*
	Mean	SD	Mean	SD	
Age (years)	57.0	10.3	56.7	10.2	0.26
Age at menarche (years) ^b^	15.0	2.4	15.0	2.0	0.80
Body mass index (kg/m^2^)	23.3	3.2	23.6	3.1	0.01
E-DII score	−0.5	2.1	−0.8	2.1	<0.01
	*n*	%	*n*	%	
Sex					0.94
Men	1425	57.0	1443	56.9	
Women	1077	43.0	1095	43.1	
Marital status					<0.01
Married	2383	95.2	2335	92.0	
Unmarried/divorced/widowed	119	4.8	203	8.0	
Residence					<0.01
Urban	1612	64.4	1997	78.7	
Rural	890	35.6	541	21.3	
Educational level					<0.01
Primary school or below	789	31.5	567	22.3	
Middle school	697	27.9	616	24.3	
High school/technical school	606	24.2	678	26.7	
College or above	410	16.4	677	26.7	
Occupation					<0.01
Administrator/other white collar worker	347	13.9	463	18.2	
Blue collar worker	547	21.9	553	21.8	
Farmer/other	1608	64.3	1522	60.0	
Income (Yuan/month)					<0.01
<2000	354	14.1	317	12.5	
2001–5000	836	33.4	935	36.8	
5001–8000	737	29.5	785	30.9	
>8001	575	23.0	501	19.7	
Menopausal status ^b^					0.92
Premenopausal	301	27.9	304	27.8	
Postmenopausal	776	72.1	791	72.2	
Occupational activity					<0.01
Nonworking	316	12.6	894	35.2	
Sedentary	697	27.9	533	21.0	
Light occupation	675	27.0	600	23.6	
Moderate occupation	390	15.6	238	9.4	
Heavy activity occupation	424	16.9	273	10.8	
Household and leisure-time activities (MET-h/week)					<0.01
Median	28.9		34.3		
25th, 75th percentiles	9.0, 52.5		16.3, 55.9		
Ever smokers	978	39.1	773	30.5	<0.01
Passive smoking	682	27.3	750	29.6	0.07
Regular drinkers	450	18.0	359	14.1	<0.01
First degree relative with cancer	362	14.5	145	5.7	<0.01
History of diabetes mellitus	214	8.6	110	4.3	<0.01
History of inflammatory diseases	117	4.7	87	4.3	0.55

MET, metabolic equivalent task. ^a^ Continuous variables were evaluated using t tests or Wilcoxon rank-sum tests. Categorical variables were evaluated using Chi square tests. ^b^ Among women subgroup.

**Table 2:** Intakes of 34 food parameters according to quartile of the energy-adjusted dietary inflammatory index (E-DII) score among controls in a Chinese case-control study, 2010–2019.

Food Parameter	Quartile 1		Quartile 2		Quartile 3		Quartile 4		*p*
	Median	P_25_, P_75_	Median	P_25_, P_75_	Median	P_25_, P_75_	Median	P_25_, P_75_	
Carbohydrate (g/day)	218.2	186.9, 262.7	230.4	197.5, 298.0	255.5	204.9, 321.8	295.7	231.1, 353.2	<0.01
Protein (g/day)	67.7	55.4, 82.1	66.3	54.0, 84.0	65.9	53.9, 81.3	66.8	52.4, 82.6	0.04
Total fat (g/day)	30.9	23.5, 42.3	32.5	22.2, 43.8	35.2	24.4, 48.7	42.6	27.3, 59.3	<0.01
Fiber (g/day)	11.3	9.3, 13.6	9.8	7.8, 12.4	8.9	7.2, 11.1	7.9	6.3, 10.0	<0.01
n-3 Fatty acids (g/day)	0.9	0.6, 1.2	0.8	0.6, 1.2	0.8	0.5, 1.1	0.8	0.6, 1.1	<0.01
n-6 Fatty acids (g/day)	4.8	3.5, 6.4	4.5	3.3, 6.1	4.5	3.3, 6.1	4.8	3.4, 6.4	<0.01
Saturated fat (g/day)	7.0	4.2, 10.5	7.5	4.3, 11.2	8.2	5.0, 12.5	10.2	5.9, 15.5	<0.01
MUFA (g/day)	8.7	5.3, 14.0	9.7	5.5, 15.6	11.0	6.7, 17.0	13.7	8.0, 21.4	<0.01
PUFA (g/day)	4.7	3.0, 6.8	4.6	2.9, 6.6	4.8	3.1, 6.8	5.3	3.4, 7.5	<0.01
Cholesterol (mg/day)	332.0	235.0, 446.2	324.5	223.4, 425.4	300.0	214.8, 421.3	282.8	190.0, 404.2	<0.01
Thiamin (mg/day)	1.0	0.8, 1.2	1.0	0.8, 1.3	1.0	0.8, 1.2	1.0	0.8, 1.3	0.81
Riboflavin (mg/day)	1.0	0.8, 1.3	0.9	0.7, 1.2	0.9	0.7, 1.1	0.8	0.6, 1.1	<0.01
Niacin (mg/day)	15.5	12.5, 18.5	15.5	12.3, 19.4	15.1	12.2, 18.8	15.2	11.9, 19.4	0.56
Folic acid (ug/day)	259.8	215.7, 321.6	228.0	187.7, 290.1	219.4	175.9, 269.5	201.8	160.4, 252.6	<0.01
Vitamin B6 (mg/day)	1	0.8, 1.2	0.9	0.7, 1.1	0.8	0.7, 1.0	0.8	0.6, 1.0	<0.01
Vitamin B12 (mg/day)	2.0	1.4, 2.7	1.9	1.3, 2.6	1.8	1.3, 2.5	1.8	1.2, 2.6	<0.01
Vitamin A (RE/day)	1034.4	845.7, 1306.1	859.8	685.6, 1101.9	756.4	578.4, 946.6	557.3	420.6, 740.4	<0.01
Vitamin C (mg/day)	198.2	157.0, 245.2	161.7	126.0, 203.9	134.7	103.6, 169.6	95.4	69.8, 126.1	<0.01
Vitamin D (ug/day)	7.4	5.1, 10.4	6.4	4.3, 9.6	5.6	3.7, 8.4	4.9	3.0, 7.2	<0.01
Vitamin E (mg/day)	13.4	10.5, 17.9	11.3	8.8, 15.0	10.3	8.0, 13.6	8.5	6.4, 11.7	<0.01
β-Carotene (ug/day)	4981.2	3980.7, 6417.9	4082.0	3166.1, 5325.2	3344.0	2551.0, 4357.1	2417.8	1643.8, 3185.0	<0.01
Iron (mg/day)	18.9	16.0, 22.6	18.0	14.7, 22.0	17.4	14.4, 21.4	16.6	13.8, 20.9	<0.01
Magnesium (mg/day)	265.1	224.0, 320.2	245.6	204.3, 312.9	241.4	198.9, 292.4	232.7	189.2, 283.0	<0.01
Selenium (ug/day)	56.8	43.7, 71.6	54.9	41.6, 71.8	52.9	41.4, 69.6	52.3	39.5, 71.5	<0.01
Zinc (mg/day)	10.8	9.0, 12.7	10.6	8.8, 13.1	10.7	8.8, 13.0	10.8	8.8, 13.4	0.13
Flavan-3-ol (mg/day)	8.9	5.1, 14.6	7.3	4.3, 12.1	6.3	3.3, 10.9	4.8	2.2, 7.6	<0.01
Flavones (mg/day)	5.7	3.0, 9.2	3.4	1.8, 6.1	2.7	1.3, 4.5	1.8	0.8, 3.6	<0.01
Flavonols (mg/day)	39.1	32.0, 49.0	30.7	24.0, 40.1	25.2	19.5, 32.5	17.8	12.6, 23.6	<0.01
Flavonones (mg/day)	4.8	2.7, 9.3	3.9	2.1, 7.5	3.2	1.8, 5.3	2.2	1.2, 4.7	<0.01
Anthocyanidins (mg/day)	22.6	15.4, 33.8	19.3	12.3, 28.0	16.4	10.8, 24.0	12.6	7.7, 19.9	<0.01
Isoflavones (mg/day)	8.2	3.1, 14.7	5.3	2.3, 10.3	4.2	1.7, 8.3	3.1	1.0, 6.6	<0.01
Garlic (g/day)	3.3	0.6, 7.1	1.7	0.1, 4.8	1.6	0.5, 3.3	1.7	0.4, 3.3	<0.01
Onion (g/day)	6.7	1.1, 14.3	3.3	0.3, 9.5	3.3	1.0, 6.7	3.3	0.7, 6.7	<0.01
Pepper (g/day)	2.1	0.0, 12.5	1.2	0.0, 8.2	0.7	0.0, 6.6	0.8	0.0, 5.0	<0.01

**Table 3:** Odds ratios and 95% confidence intervals of colorectal cancer for the energy-adjusted dietary inflammatory index (E-DII) score among cases and controls in a Chinese case-control study, 2010–2019 ^a^.

	Quartile 1		Quartile 2		Quartile 3		Quartile 4		*P*_trend_	Continuous	
	OR	95% CI	OR	95% CI	OR	95% CI	OR	95% CI		OR	95% CI
**Colorectal Cancer**											
*N* (cases/controls)	524/634		590/635		660/635		728/634			2502/2538	
Crude	1		1.12	0.96, 1.32	1.26	1.07, 1.47	1.39	1.19, 1.63	<0.01	1.07	1.04, 1.10
Adjusted	1		1.10	0.92, 1.31	1.28	1.07, 1.54	1.40	1.16, 1.68	<0.01	1.07	1.03, 1.11
**Colon Cancer**											
*N* (cases/controls)	334/634		392/635		414/635		425/634			1565/2538	
Crude	1		1.17	0.98, 1.41	1.24	1.03, 1.48	1.27	1.06, 1.52	0.01	1.05	1.02, 1.08
Adjusted	1		1.14	0.93, 1.40	1.30	1.06, 1.60	1.32	1.07, 1.63	0.01	1.06	1.02, 1.10
**Rectal Cancer**											
*N* (cases/controls)	190/634		198/635		246/635		303/634			937/2538	
Crude	1		1.04	0.83, 1.31	1.29	1.04, 1.61	1.60	1.29, 1.97	<0.01	1.10	1.06, 1.14
Adjusted	1		1.03	0.79, 1.33	1.30	1.01, 1.68	1.53	1.19, 1.98	<0.01	1.09	1.04, 1.14

^a^ OR was adjusted for age, sex, marital status, residence, educational level, occupation, income, BMI, smoking status, alcohol drinking, first degree relative with cancer, history of diabetes mellitus, occupational activity, household and leisure-time activities.

**Table 4:** Odds ratios and 95% confidence intervals of colorectal cancer for the energy-adjusted dietary inflammatory index (E-DII) score stratified by selected factors in a Chinese case-control study, 2010–2019 ^a^.

	Quartile 1		Quartile 2		Quartile 3		Quartile 4		*P*_trend_	*P*_interaction_
	OR	95% CI	OR	95% CI	OR	95% CI	OR	95% CI		
**Sex**										0.52
Men										
*N* (cases/controls)	284/361		366/361		362/361		413/360			
Crude	1		1.29	1.04, 1.59	1.28	1.03, 1.58	1.46	1.18, 1.80	<0.01	
Adjusted	1		1.33	1.04, 1.70	1.41	1.10, 1.81	1.74	1.35, 2.24	<0.01	
Women										
*N* (cases/controls)	224/274		240/274		286/274		327/273			
Crude	1		1.07	0.83, 1.37	1.28	1.00, 1.63	1.47	1.15, 1.86	<0.01	
Adjusted	1		1.05	0.80, 1.38	1.18	0.90, 1.55	1.28	0.98, 1.71	0.04	
**BMI (kg/m^2^)**										0.03
Underweight (<18.5)										
*N* (cases/controls)	30/30		34/30		50/30		45/29			
Crude	1		1.13	0.56, 2.29	1.67	0.85, 3.29	1.55	0.78, 3.09	0.13	
Adjusted	1		1.42	0.59, 3.43	1.83	0.77, 4.37	1.56	0.63, 3.87	0.28	
Normal weight (≥18.5 and <24)										
*N* (cases/controls)	276/338		377/338		301/338		387/338			
Crude	1		1.37	1.10, 1.70	1.09	0.87, 1.36	1.40	1.13, 1.74	0.03	
Adjusted	1		1.26	0.99, 1.60	1.02	0.79, 1.32	1.32	1.02, 1.71	0.13	
Overweight/Obese (≥24)										
*N* (cases/controls)	213/266		193/268		298/267		298/266			
Crude	1		0.90	0.70, 1.16	1.39	1.09, 1.78	1.40	1.10, 1.79	<0.01	
Adjusted	1		0.96	0.72, 1.28	1.48	1.12, 1.95	1.49	1.12, 1.97	<0.01	
**Smoking Status**										0.25
Ever smokers										
*N* (cases/controls)	198/193		232/194		258/193		290/193			
Crude	1		1.17	0.89, 1.54	1.30	0.99, 1.71	1.47	1.12, 1.92	<0.01	
Adjusted	1		1.15	0.84, 1.59	1.40	1.02, 1.92	1.74	1.26, 2.39	<0.01	
Nonsmokers										
*N* (cases/controls)	339/441		381/442		390/441		414/441			
Crude	1		1.12	0.92, 1.37	1.15	0.95, 1.40	1.22	1.01, 1.48	0.05	
Adjusted	1		1.14	0.92, 1.42	1.22	0.98, 1.52	1.34	1.07, 1.68	0.01	

^a^ OR was adjusted for age, sex, marital status, residence, educational level, occupation, income, BMI, smoking status, alcohol drinking, first degree relative with cancer, history of diabetes mellitus, occupational activity, household and leisure-time activities.
